# Bis(μ-nitrato-κ^2^ *O*:*O*)bis­{[1,2-bis­(diiso­propyl­phosphan­yl)-1,2-dicarba-*closo*-dodeca­borane-κ^2^ *P*,*P*′]silver(I)} di­chloro­methane disolvate

**DOI:** 10.1107/S1600536814006084

**Published:** 2014-03-22

**Authors:** Liguo Yang

**Affiliations:** a Department of Chemistry, University of Science and Technology Beijing, Beijing 100083, People’s Republic of China

## Abstract

The title compound, [Ag_2_(NO_3_)_2_(C_14_H_38_B_10_P_2_)_2_]·2CH_2_Cl_2_, was synthesized by the reaction of 1,2-bis­(diiso­propyl­phosphan­yl)-1,2-dicarba-*closo*-dodeca­borane with AgNO_3_. The resulting dinuclear molecule has crystallographically imposed inversion symmetry. The diiso­propyl­phosphanyl-*closo*-carborane ligand is coordin­ated in a bidentate manner to the Ag^I^ atom through the two P atoms. The distorted tetra­hedral coordination of the metal is completed by two O atoms of two bridging nitrate anions. The separation between the two Ag^I^ atoms is 3.8913 (5) Å. C—H⋯O hydrogen bonds are observed involving the dichloromethane solvent molecule and the nitrate anion.

## Related literature   

For related structures, see: Zhang *et al.* (2006 ▶); Paavola *et al.* (2002 ▶, 2002*a* ▶,*b* ▶). For the synthesis and structure of 1,2-bis­(di­iso­propyl­phosphan­yl)-1,2-dicarba-*closo*-dodeca­borane, see: Kivekäs *et al.* (1995 ▶). 
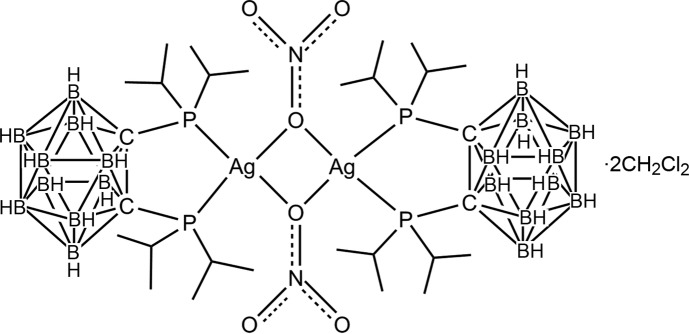


## Experimental   

### 

#### Crystal data  

[Ag_2_(NO_3_)_2_(C_14_H_38_B_10_P_2_)_2_]·2CH_2_Cl_2_
*M* *_r_* = 1262.58
Monoclinic, 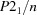

*a* = 13.9256 (15) Å
*b* = 10.3003 (10) Å
*c* = 21.8075 (19) Å
β = 107.734 (2)°
*V* = 2979.4 (5) Å^3^
*Z* = 2
Mo *K*α radiation
μ = 0.98 mm^−1^
*T* = 298 K
0.43 × 0.36 × 0.31 mm

#### Data collection  

Bruker SMART1000 CCD diffractometer
Absorption correction: multi-scan (*SADABS*; Sheldrick, 1996 ▶) *T*_min_ = 0.677, *T*_max_ = 0.751
14624 measured reflections
5264 independent reflections
3849 reflections with *I* > 2σ(*I*)
*R*_int_ = 0.027

#### Refinement  

*R*[*F* ^2^ > 2σ(*F* ^2^)] = 0.034
*wR*(*F* ^2^) = 0.091
*S* = 1.05
5264 reflections
315 parameters
6 restraints
H-atom parameters constrained
Δρ_max_ = 0.37 e Å^−3^
Δρ_min_ = −0.41 e Å^−3^

### 

Data collection: *SMART* (Siemens, 1996 ▶); cell refinement: *SAINT* (Siemens, 1996 ▶); data reduction: *SAINT*; program(s) used to solve structure: *SHELXS97* (Sheldrick, 2008 ▶); program(s) used to refine structure: *SHELXL97* (Sheldrick, 2008 ▶); molecular graphics: *SHELXTL* (Sheldrick, 2008 ▶); software used to prepare material for publication: *SHELXTL*.

## Supplementary Material

Crystal structure: contains datablock(s) I, global. DOI: 10.1107/S1600536814006084/rz5111sup1.cif

Structure factors: contains datablock(s) I. DOI: 10.1107/S1600536814006084/rz5111Isup2.hkl

CCDC reference: 992556

Additional supporting information:  crystallographic information; 3D view; checkCIF report

## Figures and Tables

**Table 1:** Hydrogen-bond geometry (Å, °)

*D*—H⋯*A*	*D*—H	H⋯*A*	*D*⋯*A*	*D*—H⋯*A*
C15—H15*A*⋯O3^i^	0.97	2.48	3.203 (6)	132
C15—H15*A*⋯O1^i^	0.97	2.48	3.389 (8)	155

Symmetry code: (i) 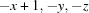.

**Fig. 1.**
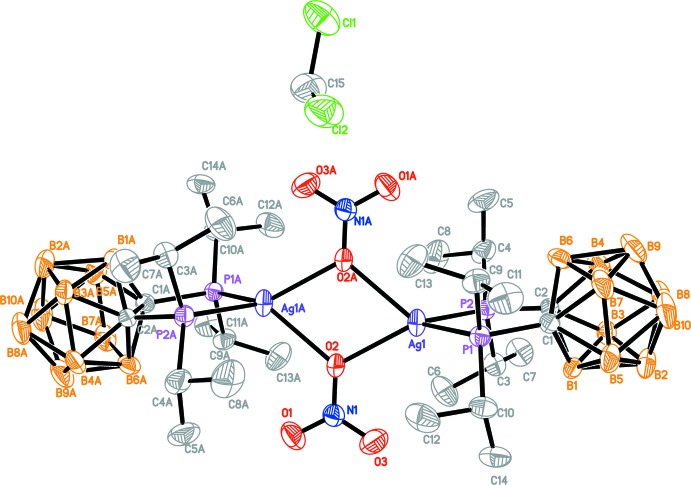
The molecular structure of the title compound with 30% probability displacement ellipsoids for non-H atoms. H atoms are omitted for clarity. Symmetry code: (A) -x + 1,-y,-z.

## supplementary crystallographic information

### 1. Comment

The synthesis and structure of 1,2-(P^i^Pr_2_)_2_-1,2-C_2_B_10_H_10_ was reported by Kivekäs *et al.* (1995). Since then, only a few complexes of this ligand with Pt(II) and Pd(II) have been described (Paavola, Kivekäs *et al.*, 2002; Paavola *et al.*, 2002**a*,b*). Here we report the structure of this ligand combined with Ag and nitrate anions.

As shown in Fig. 1, the coordination of the Ag atom is distorted tetrahedral, formed by two O atom from two NO_3_ anions and the P atoms of the diisopropylphosphanyl-*closo*-carborane ligand. The two P—Ag bond lengths are slightly shorter than the corresponding bond lengths in the complex [Ag_2_Cl_2_(C_26_H_30_B_10_P_2_)_2_].2CH_2_Cl_2_ (2.5052 (14) Å; Zhang *et al.*, 2006). The P—Ag—P angle is slightly larger than the corresponding value of 89.80 (5) Å for the complex [Ag_2_Cl_2_(C_26_H_30_B_10_P_2_)_2_].2CH_2_Cl_2_ (Zhang *et al.*, 2006). The five-memebered chelate ring formed by the silver atom, two phosphorus atoms and two carbon atoms of the carborane skeleton is strongly flattened with a maximum deviation of 0.088 (3) Å for atom C2. The torsion angle P1—C1—C2—P2 is -2.7 (3)°, *viz.* smaller than that of 12.1 (2)° in the free ligand (Kivekäs *et al.*, 1995). In the crystal, intermolecular C—H···O hydrogen bonds involving the nitrate anion and the dichloromethane solvent molecule are observed (Table 1).

### 2. Experimental

The title compound was synthesizd by the reaction of 1 mmol AgNO_3_ and 1 mmol 1,2-(P^i^Pr_2_)_2_-1,2-C_2_B_10_H_10_ in 10 ml dichloromethane under the protection of N_2_, refluxed for 4 h, then a colourless solution formed, and crystals suitable for X-ray diffraction were obtained from a dichloromethane- n-hexane (1:3 *v*/*v* solution (yield 60.7%, m.p. 553–555 K). FTIR (KBr) *v* (cm^-1^): 2989, 2966, 2930, 2872 (C—H); 2614, 2602, 2585, 2556 (B—H); 1071 (C—P).

### 3. Refinement

All H atoms were placed geometrically and treated as riding on their parent atoms, with B—H 1.10, C—H 0.96 (methyl), C—H 0.98 Å (isopropyl), with *U*_iso_(H) = 1.2*U*_eq_(B, C) or 1.5*U*_eq_(C) for methyl H atoms. A rigid bond restraints were applied to the *U*_ij_ values of atoms Ag1, P1 and P2 *via* DELU instruction of SHELXL-97 (Sheldrick, 2008).

### Crystal data

**Table:**

[Ag_2_(NO_3_)_2_(C_14_H_38_B_10_P_2_)_2_]·2CH_2_Cl_2_	*F*(000) = 1288
*M**_r_* = 1262.58	*D*_x_ = 1.407 Mg m^−^^3^
Monoclinic, *P*2_1_/*n*	Mo *K*α radiation, λ = 0.71073 Å
Hall symbol: -P 2yn	Cell parameters from 5416 reflections
*a* = 13.9256 (15) Å	θ = 2.5–26.4°
*b* = 10.3003 (10) Å	µ = 0.98 mm^−^^1^
*c* = 21.8075 (19) Å	*T* = 298 K
β = 107.734 (2)°	Block, yellow
*V* = 2979.4 (5) Å^3^	0.43 × 0.36 × 0.31 mm
*Z* = 2	

### Data collection

**Table:**

Bruker SMART1000 CCD diffractometer	5264 independent reflections
Radiation source: fine-focus sealed tube	3849 reflections with *I* > 2σ(*I*)
Graphite monochromator	*R*_int_ = 0.027
phi and ω scans	θ_max_ = 25.0°, θ_min_ = 1.6°
Absorption correction: multi-scan (*SADABS*; Sheldrick, 1996)	*h* = −13→16
*T*_min_ = 0.677, *T*_max_ = 0.751	*k* = −12→11
14624 measured reflections	*l* = −25→25

### Refinement

**Table:**

Refinement on *F*^2^	Primary atom site location: structure-invariant direct methods
Least-squares matrix: full	Secondary atom site location: difference Fourier map
*R*[*F*^2^ > 2σ(*F*^2^)] = 0.034	Hydrogen site location: inferred from neighbouring sites
*wR*(*F*^2^) = 0.091	H-atom parameters constrained
*S* = 1.05	*w* = 1/[σ^2^(*F*_o_^2^) + (0.0346*P*)^2^ + 2.5405*P*] where *P* = (*F*_o_^2^ + 2*F*_c_^2^)/3
5264 reflections	(Δ/σ)_max_ = 0.001
315 parameters	Δρ_max_ = 0.37 e Å^−^^3^
6 restraints	Δρ_min_ = −0.41 e Å^−^^3^

### Fractional atomic coordinates and isotropic or equivalent isotropic displacement parameters (Å^2^)

**Table:**

	*x*	*y*	*z*	*U*_iso_*/*U*_eq_	
Ag1	0.64127 (2)	0.05086 (3)	0.027639 (15)	0.05747 (12)	
P1	0.78280 (7)	0.00794 (9)	0.12339 (4)	0.0422 (2)	
P2	0.72744 (7)	0.21677 (9)	−0.01656 (4)	0.0446 (2)	
C1	0.8795 (2)	0.1294 (3)	0.11955 (16)	0.0440 (8)	
C9	0.7616 (3)	0.0450 (4)	0.20219 (18)	0.0593 (10)	
H9	0.7704	0.1390	0.2084	0.071*	
B1	0.9356 (3)	0.1167 (4)	0.05903 (19)	0.0444 (10)	
H1	0.9201	0.0326	0.0264	0.053*	
N1	0.5539 (3)	−0.1960 (3)	−0.05564 (16)	0.0570 (8)	
C2	0.8493 (2)	0.2413 (3)	0.04724 (16)	0.0416 (8)	
C10	0.8432 (3)	−0.1537 (4)	0.13410 (18)	0.0573 (10)	
H10	0.8990	−0.1541	0.1744	0.069*	
B2	1.0566 (3)	0.1903 (6)	0.0910 (2)	0.0707 (15)	
H2	1.1236	0.1544	0.0796	0.085*	
C3	0.7665 (3)	0.1644 (4)	−0.08742 (17)	0.0561 (10)	
H3	0.8300	0.1170	−0.0699	0.067*	
B3	0.9592 (3)	0.2734 (5)	0.0328 (2)	0.0561 (11)	
H3A	0.9641	0.2913	−0.0159	0.067*	
C11	0.8355 (4)	−0.0186 (5)	0.26173 (19)	0.0807 (14)	
H11A	0.8263	0.0183	0.3000	0.121*	
H11B	0.9034	−0.0035	0.2615	0.121*	
H11C	0.8230	−0.1103	0.2609	0.121*	
C4	0.6685 (3)	0.3796 (4)	−0.0364 (2)	0.0689 (12)	
H4	0.7133	0.4354	−0.0518	0.083*	
C5	0.6447 (4)	0.4463 (5)	0.0192 (3)	0.0957 (18)	
H5A	0.6085	0.5252	0.0042	0.143*	
H5B	0.7064	0.4659	0.0522	0.143*	
H5C	0.6042	0.3899	0.0362	0.143*	
B4	0.9034 (4)	0.3850 (5)	0.0729 (2)	0.0642 (13)	
H4A	0.8710	0.4777	0.0511	0.077*	
B5	1.0043 (3)	0.1015 (6)	0.1427 (2)	0.0632 (13)	
H5	1.0375	0.0091	0.1644	0.076*	
C12	0.7628 (4)	−0.2512 (4)	0.1399 (2)	0.0877 (15)	
H12A	0.7069	−0.2500	0.1011	0.132*	
H12B	0.7399	−0.2278	0.1757	0.132*	
H12C	0.7914	−0.3368	0.1466	0.132*	
B6	0.8485 (4)	0.2899 (4)	0.1227 (2)	0.0564 (12)	
H6	0.7772	0.3170	0.1311	0.068*	
B7	0.9491 (4)	0.2154 (5)	0.1833 (2)	0.0691 (14)	
H7	0.9461	0.1996	0.2326	0.083*	
B8	1.0358 (4)	0.3580 (6)	0.1012 (3)	0.0831 (18)	
H8	1.0901	0.4327	0.0973	0.100*	
B9	0.9660 (5)	0.3716 (6)	0.1566 (3)	0.0857 (18)	
H9A	0.9730	0.4561	0.1885	0.103*	
C6	0.6908 (4)	0.0662 (5)	−0.1280 (2)	0.0913 (16)	
H6A	0.6276	0.1087	−0.1477	0.137*	
H6B	0.6812	−0.0028	−0.1008	0.137*	
H6C	0.7162	0.0312	−0.1608	0.137*	
C7	0.7878 (4)	0.2708 (5)	−0.1299 (2)	0.0828 (14)	
H7A	0.8179	0.2334	−0.1600	0.124*	
H7B	0.8331	0.3333	−0.1036	0.124*	
H7C	0.7258	0.3128	−0.1530	0.124*	
B10	1.0634 (4)	0.2546 (7)	0.1678 (3)	0.0881 (19)	
H10A	1.1353	0.2624	0.2067	0.106*	
C13	0.6523 (4)	0.0158 (6)	0.1985 (2)	0.0881 (15)	
H13A	0.6403	−0.0759	0.1932	0.132*	
H13B	0.6077	0.0611	0.1625	0.132*	
H13C	0.6403	0.0440	0.2375	0.132*	
C8	0.5700 (4)	0.3571 (6)	−0.0918 (3)	0.111 (2)	
H8A	0.5254	0.3032	−0.0769	0.166*	
H8B	0.5853	0.3150	−0.1270	0.166*	
H8C	0.5381	0.4390	−0.1060	0.166*	
C14	0.8825 (4)	−0.1983 (4)	0.0795 (2)	0.0742 (13)	
H14A	0.8987	−0.2891	0.0845	0.111*	
H14B	0.9419	−0.1498	0.0807	0.111*	
H14C	0.8317	−0.1841	0.0390	0.111*	
Cl1	0.32691 (14)	0.43727 (14)	0.21349 (8)	0.1137 (5)	
Cl2	0.42679 (13)	0.19019 (16)	0.24175 (8)	0.1185 (5)	
O2	0.52475 (19)	−0.0987 (3)	−0.03220 (15)	0.0713 (8)	
O3	0.6446 (2)	−0.2156 (3)	−0.04215 (16)	0.0875 (10)	
O1	0.4915 (3)	−0.2662 (3)	−0.09218 (17)	0.0919 (11)	
C15	0.3518 (5)	0.2913 (6)	0.1839 (2)	0.1077 (19)	
H15A	0.3848	0.3072	0.1513	0.129*	
H15B	0.2885	0.2476	0.1632	0.129*	

### Atomic displacement parameters (Å^2^)

**Table:**

	*U*^11^	*U*^22^	*U*^33^	*U*^12^	*U*^13^	*U*^23^
Ag1	0.03991 (16)	0.0667 (2)	0.0621 (2)	−0.01337 (14)	0.01007 (13)	0.00590 (16)
P1	0.0444 (5)	0.0431 (5)	0.0413 (5)	−0.0005 (4)	0.0163 (4)	0.0016 (4)
P2	0.0398 (5)	0.0418 (5)	0.0504 (5)	0.0021 (4)	0.0111 (4)	0.0074 (4)
C1	0.0428 (19)	0.048 (2)	0.0401 (18)	−0.0014 (16)	0.0118 (15)	−0.0069 (16)
C9	0.067 (3)	0.068 (3)	0.051 (2)	0.003 (2)	0.031 (2)	0.001 (2)
B1	0.034 (2)	0.052 (3)	0.048 (2)	0.0008 (18)	0.0145 (18)	−0.002 (2)
N1	0.058 (2)	0.054 (2)	0.064 (2)	−0.0071 (18)	0.0267 (18)	−0.0053 (18)
C2	0.0419 (19)	0.0362 (19)	0.050 (2)	−0.0049 (15)	0.0188 (16)	−0.0041 (16)
C10	0.074 (3)	0.044 (2)	0.054 (2)	0.010 (2)	0.019 (2)	0.0049 (18)
B2	0.039 (2)	0.097 (4)	0.072 (3)	−0.014 (3)	0.011 (2)	−0.004 (3)
C3	0.062 (2)	0.061 (3)	0.044 (2)	−0.005 (2)	0.0133 (18)	0.0022 (18)
B3	0.048 (3)	0.059 (3)	0.066 (3)	−0.015 (2)	0.025 (2)	−0.002 (2)
C11	0.101 (4)	0.100 (4)	0.043 (2)	0.009 (3)	0.024 (2)	0.005 (2)
C4	0.059 (3)	0.056 (3)	0.094 (3)	0.017 (2)	0.027 (2)	0.022 (2)
C5	0.091 (4)	0.064 (3)	0.150 (5)	0.035 (3)	0.062 (4)	0.021 (3)
B4	0.075 (3)	0.043 (3)	0.082 (3)	−0.022 (2)	0.034 (3)	−0.013 (2)
B5	0.039 (2)	0.086 (4)	0.054 (3)	−0.002 (2)	−0.001 (2)	−0.001 (3)
C12	0.130 (5)	0.050 (3)	0.095 (4)	−0.010 (3)	0.052 (3)	0.010 (3)
B6	0.072 (3)	0.047 (3)	0.056 (3)	−0.009 (2)	0.028 (2)	−0.017 (2)
B7	0.067 (3)	0.088 (4)	0.048 (3)	−0.026 (3)	0.010 (2)	−0.022 (3)
B8	0.070 (3)	0.094 (4)	0.083 (4)	−0.045 (3)	0.020 (3)	−0.019 (3)
B9	0.102 (5)	0.080 (4)	0.077 (4)	−0.045 (4)	0.029 (3)	−0.038 (3)
C6	0.116 (4)	0.088 (4)	0.062 (3)	−0.031 (3)	0.015 (3)	−0.014 (3)
C7	0.102 (4)	0.089 (4)	0.063 (3)	−0.010 (3)	0.033 (3)	0.014 (3)
B10	0.062 (3)	0.121 (5)	0.070 (3)	−0.040 (3)	0.003 (3)	−0.018 (3)
C13	0.079 (3)	0.123 (4)	0.079 (3)	0.004 (3)	0.049 (3)	0.011 (3)
C8	0.073 (3)	0.118 (5)	0.124 (5)	0.029 (3)	0.004 (3)	0.047 (4)
C14	0.097 (4)	0.046 (2)	0.087 (3)	0.015 (2)	0.039 (3)	−0.001 (2)
Cl1	0.1405 (14)	0.0763 (9)	0.1027 (10)	−0.0029 (9)	0.0049 (9)	0.0015 (8)
Cl2	0.1154 (12)	0.1009 (11)	0.1187 (12)	0.0243 (9)	0.0050 (9)	−0.0136 (9)
O2	0.0384 (14)	0.0748 (18)	0.101 (2)	−0.0155 (11)	0.0213 (11)	−0.0315 (15)
O3	0.066 (2)	0.099 (3)	0.097 (2)	0.0177 (18)	0.0253 (18)	−0.013 (2)
O1	0.093 (2)	0.085 (2)	0.101 (2)	−0.035 (2)	0.035 (2)	−0.040 (2)
C15	0.107 (4)	0.136 (5)	0.070 (3)	0.010 (4)	0.014 (3)	−0.028 (3)

### Geometric parameters (Å, º)

**Table:**

Ag1—O2	2.328 (3)	C4—C8	1.545 (6)
Ag1—O2^i^	2.395 (3)	C4—H4	0.9800
Ag1—P1	2.4357 (9)	C5—H5A	0.9600
Ag1—P2	2.4482 (10)	C5—H5B	0.9600
P1—C10	1.848 (4)	C5—H5C	0.9600
P1—C1	1.859 (4)	B4—B9	1.773 (8)
P1—C9	1.869 (4)	B4—B8	1.779 (8)
P2—C2	1.855 (3)	B4—B6	1.797 (7)
P2—C4	1.860 (4)	B4—H4A	1.1000
P2—C3	1.867 (4)	B5—B7	1.779 (7)
C1—B5	1.679 (5)	B5—B10	1.786 (8)
C1—B7	1.684 (5)	B5—H5	1.1000
C1—B6	1.716 (6)	C12—H12A	0.9600
C1—B1	1.731 (5)	C12—H12B	0.9600
C1—C2	1.894 (5)	C12—H12C	0.9600
C9—C13	1.530 (6)	B6—B7	1.782 (7)
C9—C11	1.536 (6)	B6—B9	1.788 (7)
C9—H9	0.9800	B6—H6	1.1000
B1—C2	1.722 (5)	B7—B9	1.752 (8)
B1—B3	1.776 (6)	B7—B10	1.771 (8)
B1—B2	1.785 (6)	B7—H7	1.1000
B1—B5	1.791 (6)	B8—B10	1.748 (9)
B1—H1	1.1000	B8—B9	1.772 (8)
N1—O1	1.221 (4)	B8—H8	1.1000
N1—O3	1.223 (4)	B9—B10	1.775 (9)
N1—O2	1.248 (4)	B9—H9A	1.1000
C2—B4	1.678 (5)	C6—H6A	0.9600
C2—B3	1.686 (5)	C6—H6B	0.9600
C2—B6	1.723 (5)	C6—H6C	0.9600
C10—C14	1.525 (5)	C7—H7A	0.9600
C10—C12	1.537 (6)	C7—H7B	0.9600
C10—H10	0.9800	C7—H7C	0.9600
B2—B3	1.771 (7)	B10—H10A	1.1000
B2—B5	1.772 (7)	C13—H13A	0.9600
B2—B8	1.776 (9)	C13—H13B	0.9600
B2—B10	1.777 (8)	C13—H13C	0.9600
B2—H2	1.1000	C8—H8A	0.9600
C3—C7	1.522 (5)	C8—H8B	0.9600
C3—C6	1.532 (6)	C8—H8C	0.9600
C3—H3	0.9800	C14—H14A	0.9600
B3—B4	1.761 (7)	C14—H14B	0.9600
B3—B8	1.776 (7)	C14—H14C	0.9600
B3—H3A	1.1000	Cl1—C15	1.713 (6)
C11—H11A	0.9600	Cl2—C15	1.721 (6)
C11—H11B	0.9600	O2—Ag1^i^	2.395 (3)
C11—H11C	0.9600	C15—H15A	0.9700
C4—C5	1.514 (6)	C15—H15B	0.9700
			
O2—Ag1—O2^i^	69.06 (12)	B8—B4—B6	106.9 (4)
O2—Ag1—P1	127.18 (8)	C2—B4—H4A	122.6
O2^i^—Ag1—P1	122.75 (8)	B3—B4—H4A	122.5
O2—Ag1—P2	125.65 (8)	B9—B4—H4A	121.4
O2^i^—Ag1—P2	117.89 (8)	B8—B4—H4A	122.1
P1—Ag1—P2	95.65 (3)	B6—B4—H4A	122.7
C10—P1—C1	107.71 (17)	C1—B5—B2	108.0 (3)
C10—P1—C9	105.13 (18)	C1—B5—B7	58.2 (2)
C1—P1—C9	102.90 (17)	B2—B5—B7	107.5 (4)
C10—P1—Ag1	119.34 (13)	C1—B5—B10	106.2 (4)
C1—P1—Ag1	104.33 (11)	B2—B5—B10	59.9 (3)
C9—P1—Ag1	115.98 (13)	B7—B5—B10	59.6 (3)
C2—P2—C4	106.85 (18)	C1—B5—B1	59.7 (2)
C2—P2—C3	103.26 (16)	B2—B5—B1	60.1 (2)
C4—P2—C3	106.71 (19)	B7—B5—B1	105.8 (3)
C2—P2—Ag1	104.24 (11)	B10—B5—B1	106.8 (4)
C4—P2—Ag1	119.11 (14)	C1—B5—H5	122.6
C3—P2—Ag1	115.17 (13)	B2—B5—H5	121.3
B5—C1—B7	63.9 (3)	B7—B5—H5	123.0
B5—C1—B6	113.8 (3)	B10—B5—H5	122.5
B7—C1—B6	63.2 (3)	B1—B5—H5	122.5
B5—C1—B1	63.3 (2)	C10—C12—H12A	109.5
B7—C1—B1	113.0 (3)	C10—C12—H12B	109.5
B6—C1—B1	106.3 (3)	H12A—C12—H12B	109.5
B5—C1—P1	124.6 (3)	C10—C12—H12C	109.5
B7—C1—P1	124.0 (3)	H12A—C12—H12C	109.5
B6—C1—P1	116.9 (3)	H12B—C12—H12C	109.5
B1—C1—P1	119.0 (2)	C1—B6—C2	66.8 (2)
B5—C1—C2	107.6 (3)	C1—B6—B7	57.5 (2)
B7—C1—C2	107.1 (3)	C2—B6—B7	110.5 (3)
B6—C1—C2	56.8 (2)	C1—B6—B9	105.1 (4)
B1—C1—C2	56.53 (19)	C2—B6—B9	105.1 (3)
P1—C1—C2	117.8 (2)	B7—B6—B9	58.8 (3)
C13—C9—C11	111.1 (4)	C1—B6—B4	110.0 (3)
C13—C9—P1	110.2 (3)	C2—B6—B4	56.9 (2)
C11—C9—P1	115.9 (3)	B7—B6—B4	107.5 (4)
C13—C9—H9	106.3	B9—B6—B4	59.3 (3)
C11—C9—H9	106.3	C1—B6—H6	120.2
P1—C9—H9	106.3	C2—B6—H6	120.2
C2—B1—C1	66.5 (2)	B7—B6—H6	122.1
C2—B1—B3	57.6 (2)	B9—B6—H6	124.8
C1—B1—B3	110.3 (3)	B4—B6—H6	122.4
C2—B1—B2	105.9 (3)	C1—B7—B9	108.1 (4)
C1—B1—B2	105.1 (3)	C1—B7—B10	106.6 (3)
B3—B1—B2	59.6 (3)	B9—B7—B10	60.5 (3)
C2—B1—B5	110.4 (3)	C1—B7—B5	57.9 (2)
C1—B1—B5	56.9 (2)	B9—B7—B5	108.5 (4)
B3—B1—B5	108.3 (3)	B10—B7—B5	60.4 (3)
B2—B1—B5	59.4 (3)	C1—B7—B6	59.3 (2)
C2—B1—H1	120.0	B9—B7—B6	60.8 (3)
C1—B1—H1	120.6	B10—B7—B6	108.0 (4)
B3—B1—H1	121.7	B5—B7—B6	106.0 (3)
B2—B1—H1	124.3	C1—B7—H7	123.0
B5—B1—H1	122.0	B9—B7—H7	120.7
O1—N1—O3	122.5 (4)	B10—B7—H7	121.6
O1—N1—O2	119.1 (4)	B5—B7—H7	122.7
O3—N1—O2	118.3 (3)	B6—B7—H7	122.2
B4—C2—B3	63.1 (3)	B10—B8—B9	60.6 (4)
B4—C2—B1	112.7 (3)	B10—B8—B3	108.9 (4)
B3—C2—B1	62.8 (2)	B9—B8—B3	107.3 (3)
B4—C2—B6	63.8 (3)	B10—B8—B2	60.6 (3)
B3—C2—B6	113.1 (3)	B9—B8—B2	107.9 (4)
B1—C2—B6	106.4 (3)	B3—B8—B2	59.8 (3)
B4—C2—P2	125.8 (3)	B10—B8—B4	109.1 (4)
B3—C2—P2	124.2 (3)	B9—B8—B4	59.9 (3)
B1—C2—P2	116.6 (2)	B3—B8—B4	59.4 (3)
B6—C2—P2	119.0 (2)	B2—B8—B4	107.5 (3)
B4—C2—C1	107.2 (3)	B10—B8—H8	120.5
B3—C2—C1	106.9 (3)	B9—B8—H8	121.9
B1—C2—C1	57.0 (2)	B3—B8—H8	122.0
B6—C2—C1	56.4 (2)	B2—B8—H8	121.9
P2—C2—C1	117.5 (2)	B4—B8—H8	121.8
C14—C10—C12	108.2 (3)	B7—B9—B8	108.1 (4)
C14—C10—P1	115.5 (3)	B7—B9—B4	109.9 (3)
C12—C10—P1	106.5 (3)	B8—B9—B4	60.2 (3)
C14—C10—H10	108.8	B7—B9—B10	60.3 (3)
C12—C10—H10	108.8	B8—B9—B10	59.0 (4)
P1—C10—H10	108.8	B4—B9—B10	108.1 (4)
B3—B2—B5	109.4 (3)	B7—B9—B6	60.4 (3)
B3—B2—B8	60.1 (3)	B8—B9—B6	107.6 (3)
B5—B2—B8	107.9 (4)	B4—B9—B6	60.6 (3)
B3—B2—B10	107.8 (4)	B10—B9—B6	107.6 (4)
B5—B2—B10	60.4 (3)	B7—B9—H9A	120.7
B8—B2—B10	58.9 (3)	B8—B9—H9A	122.3
B3—B2—B1	59.9 (2)	B4—B9—H9A	120.7
B5—B2—B1	60.4 (2)	B10—B9—H9A	122.3
B8—B2—B1	106.9 (4)	B6—B9—H9A	121.8
B10—B2—B1	107.4 (3)	C3—C6—H6A	109.5
B3—B2—H2	121.2	C3—C6—H6B	109.5
B5—B2—H2	120.8	H6A—C6—H6B	109.5
B8—B2—H2	122.5	C3—C6—H6C	109.5
B10—B2—H2	122.3	H6A—C6—H6C	109.5
B1—B2—H2	122.2	H6B—C6—H6C	109.5
C7—C3—C6	110.6 (3)	C3—C7—H7A	109.5
C7—C3—P2	117.1 (3)	C3—C7—H7B	109.5
C6—C3—P2	110.2 (3)	H7A—C7—H7B	109.5
C7—C3—H3	106.1	C3—C7—H7C	109.5
C6—C3—H3	106.1	H7A—C7—H7C	109.5
P2—C3—H3	106.1	H7B—C7—H7C	109.5
C2—B3—B4	58.2 (2)	B8—B10—B7	108.3 (4)
C2—B3—B2	108.2 (3)	B8—B10—B9	60.4 (4)
B4—B3—B2	108.5 (4)	B7—B10—B9	59.2 (3)
C2—B3—B8	106.7 (3)	B8—B10—B2	60.5 (3)
B4—B3—B8	60.4 (3)	B7—B10—B2	107.6 (3)
B2—B3—B8	60.1 (3)	B9—B10—B2	107.7 (4)
C2—B3—B1	59.6 (2)	B8—B10—B5	108.6 (4)
B4—B3—B1	106.3 (3)	B7—B10—B5	60.0 (3)
B2—B3—B1	60.4 (3)	B9—B10—B5	107.2 (4)
B8—B3—B1	107.3 (4)	B2—B10—B5	59.7 (3)
C2—B3—H3A	122.7	B8—B10—H10A	120.9
B4—B3—H3A	122.4	B7—B10—H10A	122.0
B2—B3—H3A	120.9	B9—B10—H10A	122.3
B8—B3—H3A	122.0	B2—B10—H10A	121.9
B1—B3—H3A	122.3	B5—B10—H10A	121.9
C9—C11—H11A	109.5	C9—C13—H13A	109.5
C9—C11—H11B	109.5	C9—C13—H13B	109.5
H11A—C11—H11B	109.5	H13A—C13—H13B	109.5
C9—C11—H11C	109.5	C9—C13—H13C	109.5
H11A—C11—H11C	109.5	H13A—C13—H13C	109.5
H11B—C11—H11C	109.5	H13B—C13—H13C	109.5
C5—C4—C8	109.6 (4)	C4—C8—H8A	109.5
C5—C4—P2	114.3 (3)	C4—C8—H8B	109.5
C8—C4—P2	105.7 (3)	H8A—C8—H8B	109.5
C5—C4—H4	109.0	C4—C8—H8C	109.5
C8—C4—H4	109.0	H8A—C8—H8C	109.5
P2—C4—H4	109.0	H8B—C8—H8C	109.5
C4—C5—H5A	109.5	C10—C14—H14A	109.5
C4—C5—H5B	109.5	C10—C14—H14B	109.5
H5A—C5—H5B	109.5	H14A—C14—H14B	109.5
C4—C5—H5C	109.5	C10—C14—H14C	109.5
H5A—C5—H5C	109.5	H14A—C14—H14C	109.5
H5B—C5—H5C	109.5	H14B—C14—H14C	109.5
C2—B4—B3	58.6 (2)	N1—O2—Ag1	120.2 (2)
C2—B4—B9	107.7 (4)	N1—O2—Ag1^i^	128.4 (2)
B3—B4—B9	107.9 (4)	Ag1—O2—Ag1^i^	110.94 (12)
C2—B4—B8	106.9 (4)	Cl1—C15—Cl2	113.7 (3)
B3—B4—B8	60.2 (3)	Cl1—C15—H15A	108.8
B9—B4—B8	59.8 (3)	Cl2—C15—H15A	108.8
C2—B4—B6	59.3 (2)	Cl1—C15—H15B	108.8
B3—B4—B6	106.1 (3)	Cl2—C15—H15B	108.8
B9—B4—B6	60.1 (3)	H15A—C15—H15B	107.7
			
O2—Ag1—P1—C10	28.73 (18)	P1—C1—B6—C2	−107.1 (2)
O2^i^—Ag1—P1—C10	116.12 (17)	B5—C1—B6—B7	−40.4 (3)
P2—Ag1—P1—C10	−115.38 (15)	B1—C1—B6—B7	−107.9 (3)
O2—Ag1—P1—C1	148.98 (14)	P1—C1—B6—B7	116.5 (3)
O2^i^—Ag1—P1—C1	−123.63 (14)	C2—C1—B6—B7	−136.4 (3)
P2—Ag1—P1—C1	4.87 (12)	B5—C1—B6—B9	−4.2 (4)
O2—Ag1—P1—C9	−98.64 (18)	B7—C1—B6—B9	36.2 (3)
O2^i^—Ag1—P1—C9	−11.25 (18)	B1—C1—B6—B9	−71.8 (4)
P2—Ag1—P1—C9	117.25 (15)	P1—C1—B6—B9	152.7 (3)
O2—Ag1—P2—C2	−151.07 (14)	C2—C1—B6—B9	−100.3 (3)
O2^i^—Ag1—P2—C2	125.72 (13)	B5—C1—B6—B4	58.1 (4)
P1—Ag1—P2—C2	−6.15 (11)	B7—C1—B6—B4	98.5 (4)
O2—Ag1—P2—C4	90.0 (2)	B1—C1—B6—B4	−9.4 (4)
O2^i^—Ag1—P2—C4	6.79 (19)	P1—C1—B6—B4	−145.0 (3)
P1—Ag1—P2—C4	−125.08 (17)	C2—C1—B6—B4	−38.0 (3)
O2—Ag1—P2—C3	−38.70 (17)	B4—C2—B6—C1	−136.4 (3)
O2^i^—Ag1—P2—C3	−121.91 (16)	B3—C2—B6—C1	−95.6 (3)
P1—Ag1—P2—C3	106.22 (14)	B1—C2—B6—C1	−28.7 (3)
C10—P1—C1—B5	−15.7 (3)	P2—C2—B6—C1	105.4 (3)
C9—P1—C1—B5	95.0 (3)	B4—C2—B6—B7	−98.0 (4)
Ag1—P1—C1—B5	−143.5 (3)	B3—C2—B6—B7	−57.2 (4)
C10—P1—C1—B7	−95.4 (3)	B1—C2—B6—B7	9.7 (4)
C9—P1—C1—B7	15.4 (4)	P2—C2—B6—B7	143.8 (3)
Ag1—P1—C1—B7	136.8 (3)	C1—C2—B6—B7	38.4 (3)
C10—P1—C1—B6	−169.9 (3)	B4—C2—B6—B9	−36.1 (4)
C9—P1—C1—B6	−59.1 (3)	B3—C2—B6—B9	4.7 (4)
Ag1—P1—C1—B6	62.4 (3)	B1—C2—B6—B9	71.5 (4)
C10—P1—C1—B1	60.3 (3)	P2—C2—B6—B9	−154.3 (3)
C9—P1—C1—B1	171.0 (3)	C1—C2—B6—B9	100.2 (4)
Ag1—P1—C1—B1	−67.5 (3)	B3—C2—B6—B4	40.8 (3)
C10—P1—C1—C2	125.4 (2)	B1—C2—B6—B4	107.7 (3)
C9—P1—C1—C2	−123.8 (2)	P2—C2—B6—B4	−118.2 (3)
Ag1—P1—C1—C2	−2.3 (2)	C1—C2—B6—B4	136.4 (3)
C10—P1—C9—C13	−99.3 (3)	C2—B4—B6—C1	42.5 (3)
C1—P1—C9—C13	148.0 (3)	B3—B4—B6—C1	5.7 (4)
Ag1—P1—C9—C13	34.8 (4)	B9—B4—B6—C1	−96.0 (4)
C10—P1—C9—C11	27.9 (4)	B8—B4—B6—C1	−57.4 (4)
C1—P1—C9—C11	−84.7 (4)	B3—B4—B6—C2	−36.8 (3)
Ag1—P1—C9—C11	162.1 (3)	B9—B4—B6—C2	−138.5 (4)
B5—C1—B1—C2	−137.5 (3)	B8—B4—B6—C2	−99.9 (4)
B7—C1—B1—C2	−95.9 (3)	C2—B4—B6—B7	103.5 (3)
B6—C1—B1—C2	−28.6 (3)	B3—B4—B6—B7	66.7 (4)
P1—C1—B1—C2	105.9 (3)	B9—B4—B6—B7	−35.0 (4)
B5—C1—B1—B3	−99.1 (3)	B8—B4—B6—B7	3.6 (4)
B7—C1—B1—B3	−57.5 (4)	C2—B4—B6—B9	138.5 (4)
B6—C1—B1—B3	9.7 (4)	B3—B4—B6—B9	101.7 (4)
P1—C1—B1—B3	144.2 (3)	B8—B4—B6—B9	38.6 (4)
C2—C1—B1—B3	38.3 (2)	B5—C1—B7—B9	101.0 (4)
B5—C1—B1—B2	−36.4 (3)	B6—C1—B7—B9	−37.7 (4)
B7—C1—B1—B2	5.2 (4)	B1—C1—B7—B9	59.6 (5)
B6—C1—B1—B2	72.5 (4)	P1—C1—B7—B9	−143.4 (3)
P1—C1—B1—B2	−153.1 (3)	C2—C1—B7—B9	−0.6 (4)
C2—C1—B1—B2	101.1 (3)	B5—C1—B7—B10	37.3 (4)
B7—C1—B1—B5	41.6 (4)	B6—C1—B7—B10	−101.4 (4)
B6—C1—B1—B5	108.9 (3)	B1—C1—B7—B10	−4.1 (5)
P1—C1—B1—B5	−116.7 (3)	P1—C1—B7—B10	152.9 (3)
C2—C1—B1—B5	137.5 (3)	C2—C1—B7—B10	−64.3 (4)
C1—B1—C2—B4	96.4 (3)	B6—C1—B7—B5	−138.7 (3)
B3—B1—C2—B4	−40.1 (3)	B1—C1—B7—B5	−41.3 (3)
B2—B1—C2—B4	−3.6 (4)	P1—C1—B7—B5	115.6 (3)
B5—B1—C2—B4	59.2 (4)	C2—C1—B7—B5	−101.6 (3)
C1—B1—C2—B3	136.4 (3)	B5—C1—B7—B6	138.7 (3)
B2—B1—C2—B3	36.5 (3)	B1—C1—B7—B6	97.3 (3)
B5—B1—C2—B3	99.2 (3)	P1—C1—B7—B6	−105.7 (3)
C1—B1—C2—B6	28.5 (3)	C2—C1—B7—B6	37.1 (3)
B3—B1—C2—B6	−108.0 (3)	B2—B5—B7—C1	100.7 (3)
B2—B1—C2—B6	−71.4 (3)	B10—B5—B7—C1	138.1 (4)
B5—B1—C2—B6	−8.7 (4)	B1—B5—B7—C1	37.6 (3)
C1—B1—C2—P2	−106.9 (2)	C1—B5—B7—B9	−100.1 (4)
B3—B1—C2—P2	116.7 (3)	B2—B5—B7—B9	0.6 (4)
B2—B1—C2—P2	153.2 (3)	B10—B5—B7—B9	38.0 (4)
B5—B1—C2—P2	−144.1 (3)	B1—B5—B7—B9	−62.5 (4)
B3—B1—C2—C1	−136.4 (3)	C1—B5—B7—B10	−138.1 (4)
B2—B1—C2—C1	−99.9 (3)	B2—B5—B7—B10	−37.4 (3)
B5—B1—C2—C1	−37.2 (3)	B1—B5—B7—B10	−100.5 (4)
C4—P2—C2—B4	−9.0 (4)	C1—B5—B7—B6	−36.2 (3)
C3—P2—C2—B4	103.3 (3)	B2—B5—B7—B6	64.5 (4)
Ag1—P2—C2—B4	−136.0 (3)	B10—B5—B7—B6	101.9 (4)
C4—P2—C2—B3	−88.4 (3)	B1—B5—B7—B6	1.5 (4)
C3—P2—C2—B3	23.9 (3)	C2—B6—B7—C1	−42.6 (3)
Ag1—P2—C2—B3	144.6 (3)	B9—B6—B7—C1	−138.2 (4)
C4—P2—C2—B1	−162.3 (3)	B4—B6—B7—C1	−103.0 (3)
C3—P2—C2—B1	−49.9 (3)	C1—B6—B7—B9	138.2 (4)
Ag1—P2—C2—B1	70.7 (2)	C2—B6—B7—B9	95.6 (4)
C4—P2—C2—B6	68.1 (3)	B4—B6—B7—B9	35.2 (3)
C3—P2—C2—B6	−179.6 (3)	C1—B6—B7—B10	99.0 (4)
Ag1—P2—C2—B6	−58.9 (3)	C2—B6—B7—B10	56.5 (4)
C4—P2—C2—C1	133.0 (2)	B9—B6—B7—B10	−39.2 (4)
C3—P2—C2—C1	−114.7 (2)	B4—B6—B7—B10	−4.0 (4)
Ag1—P2—C2—C1	6.0 (2)	C1—B6—B7—B5	35.6 (3)
B5—C1—C2—B4	−67.0 (4)	C2—B6—B7—B5	−7.0 (4)
B7—C1—C2—B4	0.3 (4)	B9—B6—B7—B5	−102.6 (4)
B6—C1—C2—B4	40.4 (3)	B4—B6—B7—B5	−67.4 (4)
B1—C1—C2—B4	−106.3 (3)	C2—B3—B8—B10	64.8 (5)
P1—C1—C2—B4	145.8 (3)	B4—B3—B8—B10	101.5 (4)
B5—C1—C2—B3	−0.5 (4)	B2—B3—B8—B10	−36.9 (4)
B7—C1—C2—B3	66.8 (3)	B1—B3—B8—B10	2.2 (5)
B6—C1—C2—B3	106.8 (3)	C2—B3—B8—B9	0.8 (5)
B1—C1—C2—B3	−39.9 (3)	B4—B3—B8—B9	37.4 (4)
P1—C1—C2—B3	−147.8 (2)	B2—B3—B8—B9	−101.0 (5)
B5—C1—C2—B1	39.3 (3)	B1—B3—B8—B9	−61.9 (5)
B7—C1—C2—B1	106.6 (3)	C2—B3—B8—B2	101.7 (4)
B6—C1—C2—B1	146.7 (3)	B4—B3—B8—B2	138.4 (4)
P1—C1—C2—B1	−107.9 (3)	B1—B3—B8—B2	39.1 (3)
B5—C1—C2—B6	−107.4 (3)	C2—B3—B8—B4	−36.6 (3)
B7—C1—C2—B6	−40.1 (3)	B2—B3—B8—B4	−138.4 (4)
B1—C1—C2—B6	−146.7 (3)	B1—B3—B8—B4	−99.3 (3)
P1—C1—C2—B6	105.4 (3)	B3—B2—B8—B10	139.3 (4)
B5—C1—C2—P2	144.6 (3)	B5—B2—B8—B10	36.8 (3)
B7—C1—C2—P2	−148.1 (3)	B1—B2—B8—B10	100.5 (4)
B6—C1—C2—P2	−108.0 (3)	B3—B2—B8—B9	100.1 (4)
B1—C1—C2—P2	105.3 (3)	B5—B2—B8—B9	−2.4 (5)
P1—C1—C2—P2	−2.7 (3)	B10—B2—B8—B9	−39.2 (4)
C1—P1—C10—C14	−62.2 (3)	B1—B2—B8—B9	61.3 (5)
C9—P1—C10—C14	−171.4 (3)	B5—B2—B8—B3	−102.5 (3)
Ag1—P1—C10—C14	56.3 (4)	B10—B2—B8—B3	−139.3 (4)
C1—P1—C10—C12	177.7 (3)	B1—B2—B8—B3	−38.8 (3)
C9—P1—C10—C12	68.5 (3)	B3—B2—B8—B4	36.8 (3)
Ag1—P1—C10—C12	−63.8 (3)	B5—B2—B8—B4	−65.6 (4)
C2—B1—B2—B3	−35.6 (3)	B10—B2—B8—B4	−102.4 (4)
C1—B1—B2—B3	−105.0 (3)	B1—B2—B8—B4	−2.0 (5)
B5—B1—B2—B3	−140.2 (4)	C2—B4—B8—B10	−64.3 (5)
C2—B1—B2—B5	104.6 (3)	B3—B4—B8—B10	−101.1 (4)
C1—B1—B2—B5	35.3 (3)	B9—B4—B8—B10	36.8 (4)
B3—B1—B2—B5	140.2 (4)	B6—B4—B8—B10	−2.0 (5)
C2—B1—B2—B8	3.3 (4)	C2—B4—B8—B9	−101.1 (4)
C1—B1—B2—B8	−66.1 (4)	B3—B4—B8—B9	−137.9 (4)
B3—B1—B2—B8	38.9 (3)	B6—B4—B8—B9	−38.8 (3)
B5—B1—B2—B8	−101.4 (4)	C2—B4—B8—B3	36.9 (3)
C2—B1—B2—B10	65.2 (4)	B9—B4—B8—B3	137.9 (4)
C1—B1—B2—B10	−4.1 (5)	B6—B4—B8—B3	99.2 (3)
B3—B1—B2—B10	100.8 (4)	C2—B4—B8—B2	−0.1 (5)
B5—B1—B2—B10	−39.4 (4)	B3—B4—B8—B2	−37.0 (3)
C2—P2—C3—C7	−86.8 (3)	B9—B4—B8—B2	100.9 (4)
C4—P2—C3—C7	25.7 (4)	B6—B4—B8—B2	62.2 (4)
Ag1—P2—C3—C7	160.3 (3)	C1—B7—B9—B8	−63.4 (5)
C2—P2—C3—C6	145.7 (3)	B10—B7—B9—B8	35.9 (4)
C4—P2—C3—C6	−101.9 (3)	B5—B7—B9—B8	−2.1 (5)
Ag1—P2—C3—C6	32.8 (3)	B6—B7—B9—B8	−100.4 (4)
B1—C2—B3—B4	−138.3 (3)	C1—B7—B9—B4	0.7 (5)
B6—C2—B3—B4	−41.1 (3)	B10—B7—B9—B4	100.0 (4)
P2—C2—B3—B4	116.7 (3)	B5—B7—B9—B4	62.0 (5)
C1—C2—B3—B4	−101.1 (3)	B6—B7—B9—B4	−36.4 (4)
B4—C2—B3—B2	100.9 (4)	C1—B7—B9—B10	−99.3 (4)
B1—C2—B3—B2	−37.4 (3)	B5—B7—B9—B10	−38.0 (3)
B6—C2—B3—B2	59.8 (4)	B6—B7—B9—B10	−136.3 (4)
P2—C2—B3—B2	−142.4 (3)	C1—B7—B9—B6	37.0 (3)
C1—C2—B3—B2	−0.2 (4)	B10—B7—B9—B6	136.3 (4)
B4—C2—B3—B8	37.6 (4)	B5—B7—B9—B6	98.4 (3)
B1—C2—B3—B8	−100.7 (4)	B10—B8—B9—B7	−36.4 (4)
B6—C2—B3—B8	−3.5 (5)	B3—B8—B9—B7	65.8 (5)
P2—C2—B3—B8	154.3 (3)	B2—B8—B9—B7	2.8 (5)
C1—C2—B3—B8	−63.5 (4)	B4—B8—B9—B7	103.0 (4)
B4—C2—B3—B1	138.3 (3)	B10—B8—B9—B4	−139.5 (4)
B6—C2—B3—B1	97.2 (3)	B3—B8—B9—B4	−37.2 (4)
P2—C2—B3—B1	−105.1 (3)	B2—B8—B9—B4	−100.3 (4)
C1—C2—B3—B1	37.2 (2)	B3—B8—B9—B10	102.3 (4)
B5—B2—B3—C2	0.9 (5)	B2—B8—B9—B10	39.2 (4)
B8—B2—B3—C2	−99.2 (4)	B4—B8—B9—B10	139.5 (4)
B10—B2—B3—C2	−63.2 (4)	B10—B8—B9—B6	−100.3 (4)
B1—B2—B3—C2	37.0 (3)	B3—B8—B9—B6	2.0 (6)
B5—B2—B3—B4	62.5 (4)	B2—B8—B9—B6	−61.1 (5)
B8—B2—B3—B4	−37.5 (3)	B4—B8—B9—B6	39.2 (4)
B10—B2—B3—B4	−1.6 (4)	C2—B4—B9—B7	−0.5 (5)
B1—B2—B3—B4	98.7 (3)	B3—B4—B9—B7	−62.4 (5)
B5—B2—B3—B8	100.0 (4)	B8—B4—B9—B7	−100.0 (5)
B10—B2—B3—B8	35.9 (3)	B6—B4—B9—B7	36.3 (4)
B1—B2—B3—B8	136.2 (4)	C2—B4—B9—B8	99.6 (4)
B5—B2—B3—B1	−36.1 (3)	B3—B4—B9—B8	37.7 (3)
B8—B2—B3—B1	−136.2 (4)	B6—B4—B9—B8	136.3 (4)
B10—B2—B3—B1	−100.2 (4)	C2—B4—B9—B10	63.7 (4)
C1—B1—B3—C2	−42.4 (3)	B3—B4—B9—B10	1.8 (5)
B2—B1—B3—C2	−138.5 (3)	B8—B4—B9—B10	−35.9 (4)
B5—B1—B3—C2	−103.0 (3)	B6—B4—B9—B10	100.4 (4)
C2—B1—B3—B4	36.1 (3)	C2—B4—B9—B6	−36.7 (3)
C1—B1—B3—B4	−6.3 (4)	B3—B4—B9—B6	−98.6 (3)
B2—B1—B3—B4	−102.4 (4)	B8—B4—B9—B6	−136.3 (4)
B5—B1—B3—B4	−66.9 (4)	C1—B6—B9—B7	−35.6 (3)
C2—B1—B3—B2	138.5 (3)	C2—B6—B9—B7	−105.1 (3)
C1—B1—B3—B2	96.1 (3)	B4—B6—B9—B7	−140.2 (4)
B5—B1—B3—B2	35.4 (3)	C1—B6—B9—B8	65.6 (5)
C2—B1—B3—B8	99.5 (4)	C2—B6—B9—B8	−3.9 (5)
C1—B1—B3—B8	57.1 (4)	B7—B6—B9—B8	101.2 (5)
B2—B1—B3—B8	−39.0 (3)	B4—B6—B9—B8	−39.0 (4)
B5—B1—B3—B8	−3.5 (4)	C1—B6—B9—B4	104.6 (3)
C2—P2—C4—C5	−62.1 (4)	C2—B6—B9—B4	35.1 (3)
C3—P2—C4—C5	−172.1 (3)	B7—B6—B9—B4	140.2 (4)
Ag1—P2—C4—C5	55.4 (4)	C1—B6—B9—B10	3.4 (5)
C2—P2—C4—C8	177.2 (3)	C2—B6—B9—B10	−66.2 (4)
C3—P2—C4—C8	67.3 (4)	B7—B6—B9—B10	39.0 (4)
Ag1—P2—C4—C8	−65.2 (4)	B4—B6—B9—B10	−101.2 (4)
B1—C2—B4—B3	39.9 (3)	B9—B8—B10—B7	36.0 (4)
B6—C2—B4—B3	137.6 (3)	B3—B8—B10—B7	−63.7 (5)
P2—C2—B4—B3	−114.3 (3)	B2—B8—B10—B7	−100.3 (4)
C1—C2—B4—B3	100.6 (3)	B4—B8—B10—B7	−0.5 (5)
B3—C2—B4—B9	−100.6 (4)	B3—B8—B10—B9	−99.7 (4)
B1—C2—B4—B9	−60.6 (4)	B2—B8—B10—B9	−136.3 (4)
B6—C2—B4—B9	37.1 (4)	B4—B8—B10—B9	−36.5 (4)
P2—C2—B4—B9	145.2 (3)	B9—B8—B10—B2	136.3 (4)
C1—C2—B4—B9	0.1 (4)	B3—B8—B10—B2	36.6 (4)
B3—C2—B4—B8	−37.6 (3)	B4—B8—B10—B2	99.8 (4)
B1—C2—B4—B8	2.3 (4)	B9—B8—B10—B5	99.7 (4)
B6—C2—B4—B8	100.1 (4)	B3—B8—B10—B5	0.0 (6)
P2—C2—B4—B8	−151.8 (3)	B2—B8—B10—B5	−36.6 (4)
C1—C2—B4—B8	63.1 (4)	B4—B8—B10—B5	63.1 (5)
B3—C2—B4—B6	−137.6 (3)	C1—B7—B10—B8	65.2 (5)
B1—C2—B4—B6	−97.7 (3)	B9—B7—B10—B8	−36.5 (4)
P2—C2—B4—B6	108.1 (3)	B5—B7—B10—B8	101.3 (4)
C1—C2—B4—B6	−37.0 (3)	B6—B7—B10—B8	2.8 (5)
B2—B3—B4—C2	−100.4 (3)	C1—B7—B10—B9	101.7 (4)
B8—B3—B4—C2	−137.7 (4)	B5—B7—B10—B9	137.9 (4)
B1—B3—B4—C2	−36.7 (3)	B6—B7—B10—B9	39.3 (3)
C2—B3—B4—B9	100.2 (3)	C1—B7—B10—B2	1.2 (6)
B2—B3—B4—B9	−0.1 (4)	B9—B7—B10—B2	−100.5 (5)
B8—B3—B4—B9	−37.5 (3)	B5—B7—B10—B2	37.4 (4)
B1—B3—B4—B9	63.5 (4)	B6—B7—B10—B2	−61.2 (5)
C2—B3—B4—B8	137.7 (4)	C1—B7—B10—B5	−36.2 (3)
B2—B3—B4—B8	37.4 (3)	B9—B7—B10—B5	−137.9 (4)
B1—B3—B4—B8	101.0 (4)	B6—B7—B10—B5	−98.6 (3)
C2—B3—B4—B6	37.1 (3)	B7—B9—B10—B8	139.5 (4)
B2—B3—B4—B6	−63.2 (4)	B4—B9—B10—B8	36.4 (3)
B8—B3—B4—B6	−100.6 (4)	B6—B9—B10—B8	100.4 (4)
B1—B3—B4—B6	0.4 (4)	B8—B9—B10—B7	−139.5 (4)
B7—C1—B5—B2	−99.8 (4)	B4—B9—B10—B7	−103.1 (4)
B6—C1—B5—B2	−59.7 (4)	B6—B9—B10—B7	−39.0 (3)
B1—C1—B5—B2	37.3 (3)	B7—B9—B10—B2	100.3 (4)
P1—C1—B5—B2	145.5 (3)	B8—B9—B10—B2	−39.1 (3)
C2—C1—B5—B2	1.0 (4)	B4—B9—B10—B2	−2.7 (5)
B6—C1—B5—B7	40.1 (3)	B6—B9—B10—B2	61.3 (5)
B1—C1—B5—B7	137.1 (3)	B7—B9—B10—B5	37.5 (3)
P1—C1—B5—B7	−114.7 (3)	B8—B9—B10—B5	−102.0 (4)
C2—C1—B5—B7	100.8 (3)	B4—B9—B10—B5	−65.6 (5)
B7—C1—B5—B10	−36.8 (3)	B6—B9—B10—B5	−1.6 (5)
B6—C1—B5—B10	3.3 (4)	B3—B2—B10—B8	−36.4 (3)
B1—C1—B5—B10	100.3 (4)	B5—B2—B10—B8	−139.1 (4)
P1—C1—B5—B10	−151.5 (3)	B1—B2—B10—B8	−99.6 (4)
C2—C1—B5—B10	64.0 (4)	B3—B2—B10—B7	65.1 (5)
B7—C1—B5—B1	−137.1 (3)	B5—B2—B10—B7	−37.5 (4)
B6—C1—B5—B1	−97.0 (3)	B8—B2—B10—B7	101.5 (5)
P1—C1—B5—B1	108.2 (3)	B1—B2—B10—B7	1.9 (6)
C2—C1—B5—B1	−36.3 (3)	B3—B2—B10—B9	2.6 (5)
B3—B2—B5—C1	−1.2 (5)	B5—B2—B10—B9	−100.0 (4)
B8—B2—B5—C1	62.5 (4)	B8—B2—B10—B9	39.1 (4)
B10—B2—B5—C1	98.7 (4)	B1—B2—B10—B9	−60.5 (5)
B1—B2—B5—C1	−37.1 (3)	B3—B2—B10—B5	102.6 (4)
B3—B2—B5—B7	−62.6 (4)	B8—B2—B10—B5	139.1 (4)
B8—B2—B5—B7	1.2 (4)	B1—B2—B10—B5	39.4 (3)
B10—B2—B5—B7	37.3 (3)	C1—B5—B10—B8	−64.7 (5)
B1—B2—B5—B7	−98.5 (3)	B2—B5—B10—B8	37.0 (4)
B3—B2—B5—B10	−99.9 (4)	B7—B5—B10—B8	−100.9 (5)
B8—B2—B5—B10	−36.2 (4)	B1—B5—B10—B8	−2.1 (5)
B1—B2—B5—B10	−135.8 (4)	C1—B5—B10—B7	36.2 (3)
B3—B2—B5—B1	35.9 (3)	B2—B5—B10—B7	137.9 (4)
B8—B2—B5—B1	99.7 (4)	B1—B5—B10—B7	98.8 (4)
B10—B2—B5—B1	135.8 (4)	C1—B5—B10—B9	−0.9 (5)
C2—B1—B5—C1	41.4 (3)	B2—B5—B10—B9	100.8 (4)
B3—B1—B5—C1	102.8 (3)	B7—B5—B10—B9	−37.1 (4)
B2—B1—B5—C1	138.3 (4)	B1—B5—B10—B9	61.7 (4)
C2—B1—B5—B2	−96.9 (4)	C1—B5—B10—B2	−101.7 (4)
C1—B1—B5—B2	−138.3 (4)	B7—B5—B10—B2	−137.9 (4)
B3—B1—B5—B2	−35.5 (3)	B1—B5—B10—B2	−39.1 (3)
C2—B1—B5—B7	4.5 (4)	O1—N1—O2—Ag1	−172.7 (3)
C1—B1—B5—B7	−36.9 (3)	O3—N1—O2—Ag1	5.6 (5)
B3—B1—B5—B7	65.8 (4)	O1—N1—O2—Ag1^i^	16.1 (5)
B2—B1—B5—B7	101.4 (4)	O3—N1—O2—Ag1^i^	−165.6 (3)
C2—B1—B5—B10	−57.9 (4)	O2^i^—Ag1—O2—N1	−172.6 (4)
C1—B1—B5—B10	−99.3 (4)	P1—Ag1—O2—N1	−56.7 (3)
B3—B1—B5—B10	3.5 (4)	P2—Ag1—O2—N1	77.4 (3)
B2—B1—B5—B10	39.0 (4)	O2^i^—Ag1—O2—Ag1^i^	0.0
B5—C1—B6—C2	96.1 (3)	P1—Ag1—O2—Ag1^i^	115.89 (11)
B7—C1—B6—C2	136.4 (3)	P2—Ag1—O2—Ag1^i^	−109.99 (11)
B1—C1—B6—C2	28.5 (3)		

Symmetry code: (i) −*x*+1, −*y*, −*z*.

### Hydrogen-bond geometry (Å, º)

**Table:**

*D*—H···*A*	*D*—H	H···*A*	*D*···*A*	*D*—H···*A*
C15—H15*A*···O3^i^	0.97	2.48	3.203 (6)	132
C15—H15*A*···O1^i^	0.97	2.48	3.389 (8)	155

Symmetry code: (i) −*x*+1, −*y*, −*z*.
